# Supplementary material for: Statistical Conceptualisation of Mood Instability: A Systematic Review
Source: Brain Sci. 2025 Sep 29;15(10):1059. doi: 10.3390/brainsci15101059 (PMC12563519; doi:10.3390/brainsci15101059)
Supplement: Supplementary file 1 [file brainsci-15-01059-s001.zip › brainsci-3832034-supplementary.pdf]

Supplementary materials

Table S1. Full Search Terminology

1	Exp emotional instability
**2**	emotion$ instability' or 'emotion$ unstable' or emotion$ labil$' or emotion$ dysregulat$' or emotion$ variab$' or 'emotion$ fluctuat$'
**3**	'affect$ instability' or 'affect$ labil$' or 'affect$ dysregulat$' or affect$ variab$' or 'affect$ fluctuat$'
**4**	'Mood instability' or 'mood labil$' or 'mood dysregulat$' or 'mood variab$' or 'mood fluctat$'
**5**	(1 or 2 or 3 or 4)
**6**	Exp ecological momentary assessment
**7**	'experience sampling' or experience sampling method$'
**8**	mood monitoring
**9**	(6 or 7 or 8)
**10**	(5 AND 9)

Limits : English Language1950 - current

Table S2: Full List of terminology to describe Statistical Methods

Mean Squared Successive Difference	Mean Adjusted Absolute Squared Successive Difference
Standard Deviation	Aggregated point by point change
Root Mean Squared Successive Difference	Recurrence Quantification Analysis (RQA)
Squared Successive Difference	Coefficient of Variation
Autocorrelation	Intraindividual Standard Deviation
Teager-Kaiser Energy Operator (TKEO)	ACORR
Entropy	Short-Term Probability of Acute Change
Adjusted squared successive differences	Long-Term Probability of Acute Change
Probability of Acute Change	Multi Level Model
Inter-Item Standard Deviation	Fragmentation Measures
Within-Person Variance	Regression Model
Short-Term Mean Squared Successive Difference	Affect Spin
Autoregression	State Space Grids

Figure S1: Pie Chart to show use of different statistical methods described including all terminologies.

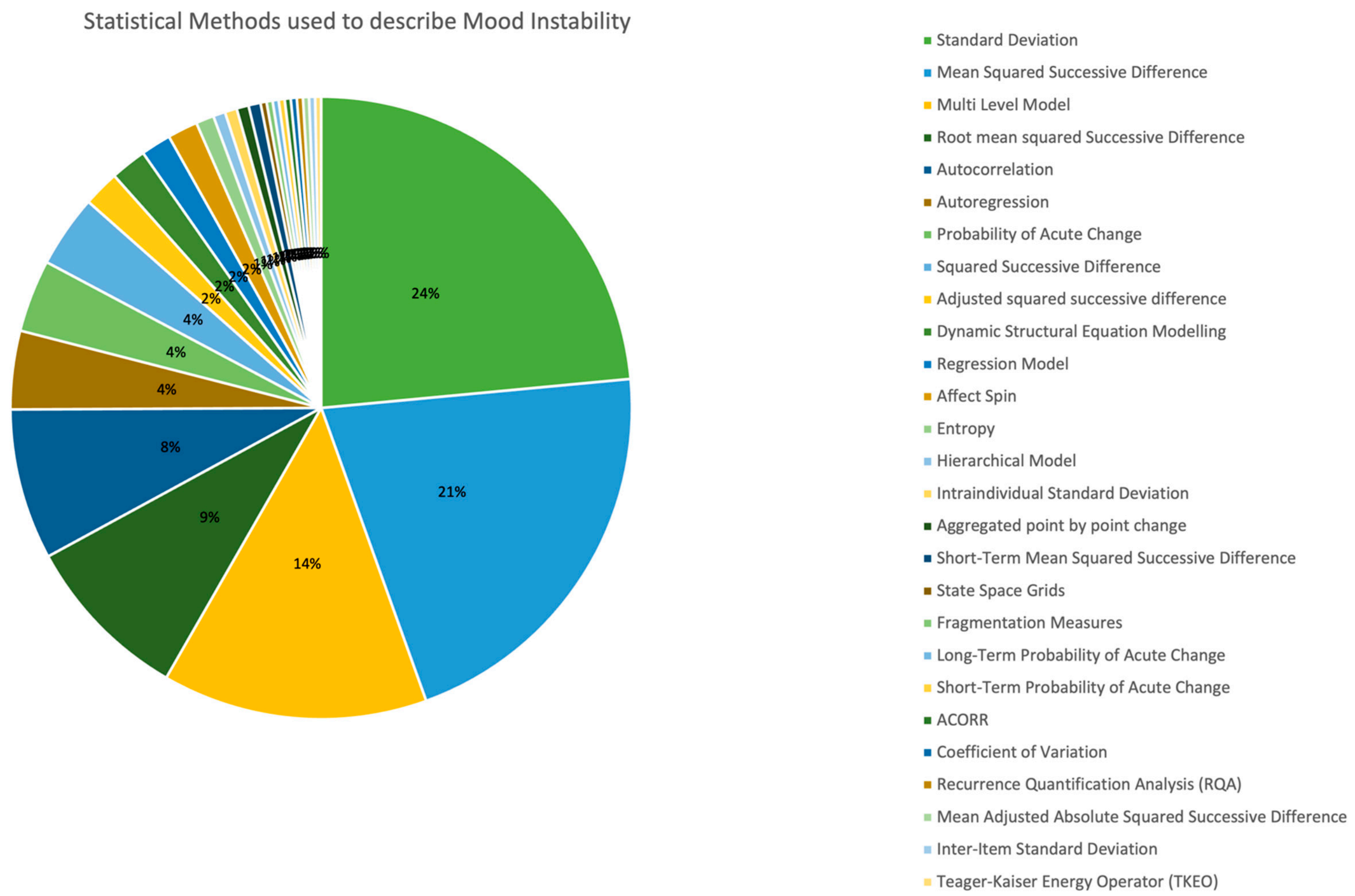

Table S3: List of Terminology used to describe different constructs of mood instability

Variability	Reactivity
Instability	flux
Inertia	Stability
Lability	Affective Home Base
Intensity	Attractor Strength
Valence	Continuity
Arousal	Affect Switch
Fluctuations	Activation
Pulse	Persistence
Spin	Differentiation
Recovery	Interaction

Chi Square Distribution tables used to deconstruct the significant effect of the Type of Sample and statistic methods used.

Table S4: Contingency Table to compare studies with clinical and non-clinical samples and their use of MSSD and SD.

Type of Sample	MSSD	SD
Non-Clinical	42	46
Clinical	27	17
Clinical and Healthy Controls	46	13

Table S5: Contingency table to compare studies with clinical and non-clinical samples and their use of SD and MLM.

	SD	MLM
Non-Clinical	46	27
Clinical	17	10
Clinical and Healthy controls	13	7
